# Lower galactosylation levels of the Lipophosphoglycan from *Leishmania (Leishmania) major*-like strains affect interaction with *Phlebotomus papatasi* and *Lutzomyia longipalpis*

**DOI:** 10.1590/0074-02760170333

**Published:** 2018-02-19

**Authors:** Agna Cristina Guimarães, Paula Monalisa Nogueira, Soraia de Oliveira Silva, Jovana Sadlova, Katerina Pruzinova, Jana Hlavacova, Maria Norma Melo, Rodrigo Pedro Soares

**Affiliations:** 1 Universidade Federal de Minas Gerais, Departamento de Parasitologia, Belo Horizonte, MG, Brasil; 2 Fundação Oswaldo Cruz-Fiocruz, Instituto René Rachou, Belo Horizonte, MG, Brasil; 3 Charles University, Faculty of Science, Department of Parasitology, Prague, Czech Republic

**Keywords:** Leishmania major, like, lipophosphoglycan, host-parasite interaction, Phlebotomus papatasi, Lutzomyia longipalpis

## Abstract

**BACKGROUND:**

*Leishmania major* is an Old World species causing cutaneous leishmaniasis and is transmitted by *Phlebotomus papatasi* and *Phlebotomus duboscqi*. In Brazil, two isolates from patients who never left the country were characterised as *L. major*-like (BH49 and BH121). Using molecular techniques, these isolates were indistinguishable from the *L. major* reference strain (FV1).

**OBJECTIVES:**

We evaluated the lipophosphoglycans (LPGs) of the strains and their behaviour in Old and New World sand fly vectors.

**METHODS:**

LPGs were purified, and repeat units were qualitatively evaluated by immunoblotting. Experimental *in vivo* infection with *L. major-*like strains was performed in *Lutzomyia longipalpis* (New World, permissive vector) and *Ph. papatasi* (Old World, restrictive or specific vector).

**FINDINGS:**

The LPGs of both strains were devoid of arabinosylated side chains, whereas the LPG of strain BH49 was more galactosylated than that of strain BH121. All strains with different levels of galactosylation in their LPGs were able to infect both vectors, exhibiting colonisation of the stomodeal valve and metacyclogenesis. The BH121 strain (less galactosylated) exhibited lower infection intensity compared to BH49 and FV1 in both vectors.

**MAIN CONCLUSIONS:**

Intraspecific variation in the LPG of *L. major*-like strains occur, and the different galactosylation levels affected interactions with the invertebrate host.

*Leishmania major* is one of the most important species causing cutaneous leishmaniasis (CL) in the Old World. It is widely distributed from Sub-Saharan Africa to the Indian subcontinent South-Central Asia. This species is transmitted by the bite of the sand flies, *Phlebotomus papatasi* and *Phlebotomus duboscqi*, depending on the region (Anderson et al. 2011, Salam et al. 2014). In several countries of South America (Brazil, Ecuador, Paraguay, Venezuela, and Mexico), many CL isolates were biochemically and molecularly identified as *L. major* and were named *L. major*-like (Momen et al. 1985, 1993, Calvopina et al. 2004, Silva et al. 2009, Hashiguchi et al. 2016).

In the 1970s, two isolates were recovered from CL patients from the Brazilian states, Goiás and Minas Gerais. The patients were rural workers who claimed they had never left the region. The strains remained frozen until further examination in the 1990s. Surprisingly, the isolates were shown to be identical to the World Health Organization reference strains, Friedlin (FV1) and 5-ASKH by isoenzyme, RAPD and SSR-PCR analysis (Silva et al. 2009). The *L. major*-like strains were named BH49 (Goiás) and BH121 (Minas Gerais). By the time they were isolated, specific epidemiological studies were not available to incriminate the vector(s) and wild host(s); however, a lot of information was available on regarding the vectors and hosts of *Leishmania braziliensis* (Mayrink et al. 1979, Rocha et al. 1988). Although both strains were isolated from human skin lesions, a distinguishing feature exhibited by the BH49 strain was its higher virulence for the murine model compared to that of BH121 (non-virulent) (Silva et al. 2009). To ascertain the possible mechanisms underlying their virulence, two virulence genes highly expressed in the BH49 strain were recently identified; one encoded a protein homologous to α-haemolysin and another encoded a protein homologous to the β-1,3-galactosyltransferase 3 (Wu et al. 2015). The latter is a transferase responsible for galactosyl residues assembly on the LPG repeat units in *L. major* (Dobson et al. 2003). These residues are important for *L. major/Leishmania turanica* attachment to the midgut receptor, PpGalec (Kamhawi et al. 2004, Volf et al. 2014). However, interactions between *L. major*-like strains bearing LPG polymorphisms with the invertebrate host were not examined.

Although these strains were genetically similar to Old World *L. major*, their glycobiology and infectivity to New and Old World vectors remain unknown. Here, we qualitatively evaluated the presence of galactose/arabinose in the LPGs of *L. major*-like strains (BH49 and BH121). We additionally evaluated their interactions with vectors *Lutzomyia longipalpis* (permissive) and *Ph. papatasi* (restrictive).

## MATERIALS AND METHODS

*Parasites and LPG purification* - World Health Reference strains of *L. major* (MHOM/IL/1980/Friedlin), *Leishmania infantum* (MCAN/BR/89/Ba-262), and *L. major*-like strains BH49 (MHOM/BR/1971/BH49) and BH121 (MHOM/BR/1971/BH121) were used. *L. major*-like strains were isolated from human CL cases in Brazil: BH49 in the State of Goiás and BH121 in the State of Minas Gerais. Frozen stocks of strains passaged in mice were characterised and typed, as previously reported (Silva et al. 2009). Promastigotes were cultured in M199 medium supplemented with 10% heat-inactivated foetal bovine serum (FBS), penicillin 100 units mL^-1^, streptomycin 50 mg mL^-1^, 12.5 mM glutamine, 0.1 M adenine, 0.0005% hemin, and 40 mM Hepes, pH 7.4 at 26ºC. LPGs from late log parasites (procyclics) were extracted in solvent E (H_2_O/ethanol/diethyl ether/pyridine/NH_4_OH, 15:15:5:1:0.017), as previously described (Soares et al. 2002). The solvent E extract was dried by N_2_ evaporation, resuspended in 0.1 N acetic acid/0.1 M NaCl, and applied to a column of phenyl-Sepharose (2 mL), equilibrated with the same buffer. LPG was eluted in solvent E, dried, and resuspended in sterile, LPS-free distilled water (Sanobiol, Campinas, Brazil).

*Immunoblotting* - Purified LPGs (10 μg) from different strains were transferred to nitrocellulose paper; the membrane was blocked in 5% milk in PBS and probed with the following antibodies (1:1,000): WIC 79.3, which recognises terminal Gal(β1,3) sequences that branch off the repeat units, and 3F12, which recognises terminal Ara(β1,2)Gal(β1,3) (Kelleher et al. 1992, 1994). For dot-blots, LPGs (5 μg) were applied to the nitrocellulose membrane; the membranes were blocked as described above and probed with CA7AE (1:1000), which recognises the Gal(β1,4)Man(α1)-PO_4_ repeat units (Tolson et al. 1989). After three washes in PBS for 10 min, membranes were incubated with anti-mouse IgG conjugated with horseradish peroxidase (1:10,000), and the reaction was visualized using Luminol (Nogueira et al. 2016).

*Sand fly colonies, experimental infections, and morphometry* - Colonies of *Lu. longipalpis* (originating from Jacobina, Brazil) and *Ph. papatasi* (originating from Cukurova, Turkey) were maintained in the insectary at the Department of Parasitology, Charles University in Prague under standard conditions (Volf & Volfova 2011). Sand fly females (2-6 d old) were fed through a chick-skin membrane on heat-inactivated rabbit blood containing 10^6^ promastigotes/mL. Reference strain of *L. major* (MHOM/IL/1980/Friedlin) was used as the control since it is the most used model for sand fly infections. Sand-flies were infected with the strains in two parallel sets of experiments using all sand-fly *Leishmania* combinations. Engorged females were separated, maintained in the same conditions as the colony, and dissected on days 1, 5, and 8 post-infection (PI). Dissected guts were divided into several parts: the abdominal midgut (AMG), thoracic midgut (TMG), stomodeal valve (SV), cardia, and endoperithrophic space. They were examined under a microscope to determine the localisation and intensity of *Leishmania* infections. Parasite loads were graded as light (< 100 parasites/gut), moderate (100-1000 parasites/gut), or heavy (> 1000 parasites/gut), as described by Myskova et al. (2008). To evaluate the morphological forms of *Leishmania*, midgut smears fixed with methanol and stained with Giemsa were examined under a light microscope with an oil-immersion objective and photographed with an Olympus D70 camera. Body length, body width, and flagellar length of 120 randomly selected promastigotes from three females/smears were measured for each sand fly species and time interval using Image-J software. The morphological forms were distinguished, according to the criteria described by Walters (1993) and Cihakova and Volf (1997). Data were evaluated statistically by means of the Fisher’s exact or Chi-square (χ^2^) tests using SPSS statistics 23 software.

## RESULTS

**Fig. 1:**
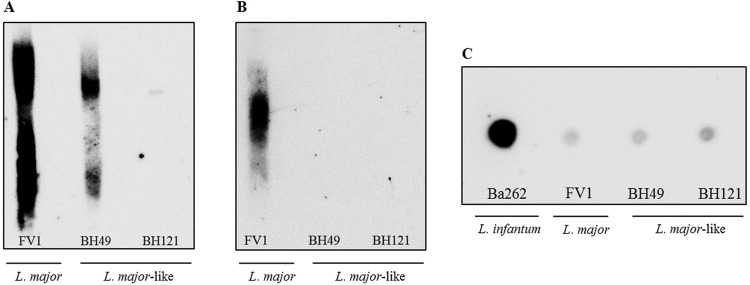
: western blotting and dot blotting analysis of purified lipophosphoglycans (LPGs). Immunoblotting of purified LPG (5-10 μg per lane) from late-log forms of *Leishmania major* (FV1), *L. major*-like, (BH49 and BH121) and *L. infatum* (Ba262) strains probed with the monoclonal antibodies WIC 79.3 (A), 3F12 (B) and CA7AE (C).

*LPGs from L. major-like strains display intraspecific polymorphisms* - For preliminary qualitative analysis, immunoblotting (western-blot and dot-blot) techniques were performed using standard mAbs specific for the repeat unit epitopes of *Leishmania* spp. By western-blot, LPGs purified from *L. major*-like strains were differentially recognised by antibody WIC 79.3, specific for terminal β-galactosyl residues. LPGs from FV1 and BH49 strains were strongly recognised by this antibody. However, the LPG from BH121 reacted very poorly with the antibody (Fig. 1A). In contrast, none of the LPGs from *L. major*-like strains were recognized by 3F12, suggesting that their repeat units were devoid of arabinose side chains (Fig. 1B). As expected, only the LPG from *L. major* (FV1) reacted with 3F12. Since the LPGs from *L. major*-like strains exhibited different levels of glycosylation in their side-chains, they were subjected to a dot-blot assay using CA7AE that recognizes unbranched repeat units. Consistent with these observations, LPGs from *L. major*-like strains and the positive *L. infantum* control (Ba262) were recognised by this antibody. Moreover, a very clear cline was observed where the lower galactosylated LPGs were more reactive for CA7AE. The pattern could be seen more clearly in the dot-blot compared to western-blot. As expected, the LPG from *L. major* (FV1) reacted poorly with this antibody (Fig. 1C). Together, these data indicate that the LPGs from *L. major*-like strains display polymorphisms in their structures. They are not arabinosylated, but have galactosylated or unbranched side-chains (Table).

**TABLE:** Lipophosphoglycan polymorphisms and sand fly infections

Strains	Repeat unit type^*^			Sand fly infection	
	
	Gal(β1,3)	Ara(β1,2)	Gal(β1,4)Man(α1)-PO_4_	*Lutzomyia longipalpis*	*Phlebotomus papatasi*
*Leishmania major* (FV1)	+++	+++	+	+++	+++
*L. major-*like (BH49)	+++	-	+	+++	+++
*L. major-*like (BH121)	-	-	++	++	++

*: based on antibody recognition.

**Fig. 2:**
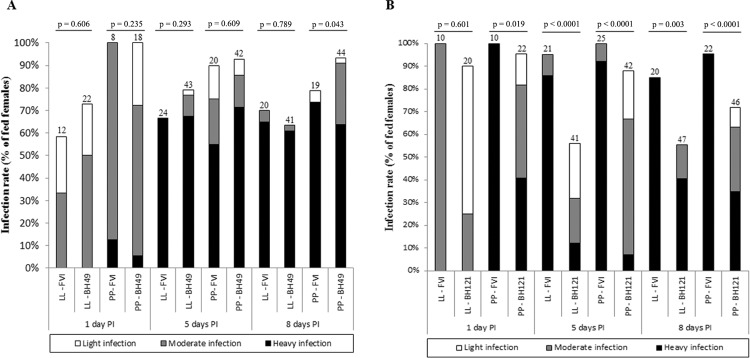
: development of *Leishmania major*-like strains BH49 (A) and BH121 (B) in *Lutzomyia longipalpis* (LL) and *Phlebotomus papatasi* (PP). Rates and intensities of infections were evaluated under a microscope on days 1, 5, and 8 PI and were classified into three categories: light (< 100 parasites/gut), moderate (100-1000 parasites/gut), or heavy (> 1000 parasites/gut). The number of dissected females is shown above the bars. P < 0.05 were considered statistically significant.

**Fig. 3:**
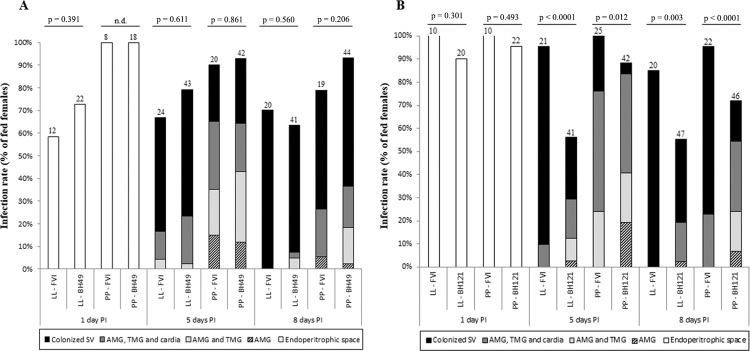
: localisation of *Leishmania major*-like strains BH49 (A) and BH121 (B) in *Lutzomyia longipalpis* (LL) and *Phlebotomus papatasi* (PP) on days 1, 5, and 8 PI. Abdominal midgut (AMG), thoracic midgut (TMG), and stomodeal valve (SV), n.d., not determined. P < 0.05 were considered statistically significant.

**Fig. 4:**
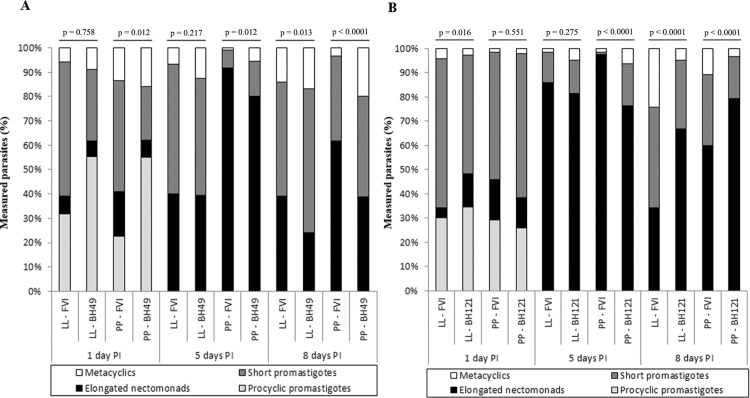
: morphological forms of *Leishmania major*-like parasites from strain BH49 (A) and BH121 (B) during development in *Lutzomyia longipalpis* (LL) and *Phlebotomus papatasi* (PP) on days 1, 5, and 8 PI. Parasites were classified by morphology as procyclic promastigotes, elongated nectomonads, short promastigotes (leptomonads), and metacyclics, as described by Walters (1993) and Cihakova and Volf (1997). The frequency (%) of each morphological form found in infected flies at each time point is shown. Differences between HB49 and BH121 strains versus *L. major* FVI strain were tested by *χ*2 test; p < 0.05 was considered statistically significant.

*Sand fly infections and morphometry of promastigotes* - Since LPGs from *L. major*-like strains displayed intraspecific variations in galactose residues; the impact of the variations on the interaction with sand flies was evaluated. All strains successfully infected *Lu. longipalpis* (LL) and *Ph. papatasi* (PP) sand flies (Figs 2-4). The intensity of infection of the BH49 strain was very similar to FV1 in both vectors, except on day 8 in *Ph. papatasi* (p < 0.05) (Fig. 2A). Interestingly, the BH121 strain infection rate was always lower in both vectors on all days (p < 0.05) (Fig. 2B). Similar to the infection intensity pattern, both strains were equally able to colonise all parts of the foregut and midgut and reached the stomodeal valve (SV) in both sand fly species (Fig. 3). Colonisation of SV by the BH49 strain was not different from FV1 (p > 0.05). The ability of the BH121 strain to reach SV was lower than that of FV1 (p < 0.05) (Fig. 3B). All expected promastigote forms were observed in both vectors with variations in the nectomonad/metacyclic density, depending on the strain (Fig. 4). For example, the FV1 strain produced a greater number of nectomonads on day 5 compared to BH49/BH121 (p < 0.01) (Fig. 4A-B). Additionally, the ability of the BH49 strain to produce metacyclics on day 8 was higher than that of FV1 in *Ph. papatasi* (p < 0.05) (Fig. 4A). Similar to the other parameters, metacyclogenesis in the strain BH121 was lower than that in FV1 on day 8 for both vectors (p < 0.05) (Fig. 4B).

## DISCUSSION

*L. major*-like strains have been isolated in several countries in Latin America, despite the fact that this species in commonly found in Old World regions. Several aspects of their epidemiology regarding vectors and reservoirs are scarce. Only two studies have addressed some biological, biochemical, and molecular parameters using *L. major*-like strains (Silva et al. 2009, Wu et al. 2015). Here, we qualitatively evaluated LPG structures in *L. major*-like strains and determined their impact during *in vivo* interaction with sand flies. LPG is the major glycoconjugate of *Leishmania*; LPG is involved in a wide range of functions (Assis et al. 2012). The LPG of *L. major* (FV1) has β-1,3 galactosyl side-chains (McConville et al. 1992) that are important in the interaction with the midgut of *Ph. papatasi* (Kamhawi et al. 2004). Similar to *L. major* (FV1)*,* BH49 exhibited β-galactosyl residues in their LPGs. An interesting feature of the BH121 strain was its absent or low galactosylation level, based on WIC 79.3 recognition. Although both strains did not show arabinosyl residues in their LPGs, these sugars are not important for attachment to the sand fly midgut and may be absent in other *L. major* strains, such as LV39 (Dobson et al. 2003). The LPGs of the strains BH49 and BH121 differed from those of FV1 strain and exhibited some repeat units devoid of side-chains when probed with mAb, CA7AE. The reactivity of the LPG of the FV1 strain to this antibody is very low or absent (Butcher et al. 1996, Volf et al. 2014) while the reactivity of the LPG of BH121 is increased; the BH121 LPG is poorly galactosylated. In conclusion, the LPGs from Brazilian *L. major*-like strains possess intraspecific polymorphisms.

In order to determine whether such variations could have an impact during development within the invertebrate host, sand flies were infected with the strains *in vivo*. Although we used recently frozen stocks isolated from mouse (Silva et al. 2009), it is already known that long term *in vitro* passaging has a negligible effect on *Leishmania* infectivity in sand flies (Cihakova & Volf 1997, Sádlová et al. 1999). Since no vector species were incriminated at the time that *L. major*-like strains were isolated, we chose one vector that was present in the area (*Lu. longipalpis*) (Mayrink et al. 1979) and the Old World vector for *L. major* (*Ph. papatasi*). In permissive vectors such as *Lu. longipalpis* and *Lu. migonei*, intraspecies variations in the LPGs of *L. infantum* and *L. amazonensis* did not affect interaction (Coelho-Finamore et al. 2011, Nogueira et al. 2016, 20, 2017). We also evaluated the interaction with a restrictive vector (*Ph. papatasi*). This vector is only susceptible to Old World *L. major* and *L. turanica* (Chajbullinova et al. 2012). In contrast with previous studies, *L. major*-like strains bearing variations in their LPG galactosylation levels differently infected both vectors. Previous reports have shown that *Lu. longipalpis* is a permissive vector able to sustain infection with different *Leishmania* species including *L. major* (reviewed by Soares & Turco 2003). Consistent with those observations, both strains and the control (FV1) successfully colonized the foregut and midgut of *Lu. longipalpis* and *Ph. papatasi*. Although both strains were able to infect both vectors and accomplish metacyclogenesis, the intensity of this infection was significantly lower in the BH121 strain. This is the first evidence that *Leishmania* strains bearing intraspecies polymorphisms in their LPG exhibited variations during the interaction with restrictive and permissive sand-flies. One of the hypotheses is that the presence of a smaller number of galactose residues in the LPG could adversely affect parasite attachment to the midgut. Since both *L. major*-like strains were able to survive and sustain infection in *Ph. papatasi*, a restrictive vector, this is a strong indication that galactosylation may be one of the factors involved. Besides, since *Ph. papatasi* is not found in Brazil, those strains may have used an alternative vector in the New World. Although *Lu. longipalpis* has urbanized in many cities in Latin America (revised by Salomon et al. 2015) and its occurrence in rural areas is rare, it could be one of the suspected vectors for transmitting *L. major*-like strains. Although *Lu. longipalpis* is a proven vector for *L. infantum*, the transmission of *L. major*-like strains by this vector and other vectors has not been observed yet. More importantly, other CL vectors in those geographical regions such as *Lu. whitmani*, *Lu. migonei* and *Lu. intermedia* should also be investigated. This work highlights the importance of returning to the field to address these questions.

In conclusion, *L. major*-like strains were able to successfully infect *Lu. longipalpis* and *Ph. papatasi.* The observed differences, among other factors, seemed to be controlled by variations in the galactose residues in the LPG. However, there are still many uncertainties regarding the origin and introduction of *L. major*-like strains in the Americas. Like *L. infantum*, this could have occurred after the introduction of infected dogs (reviewed by Mauricio et al. 2000), but the reservoir(s) of *L. major*-like strains in the Americas remain unknown. After more than 40 years, the regions from where these strains were isolated were modified by anthropic action, and the eco epidemiology of leishmaniasis may have changed completely. This reinforces the need for novel epidemiological studies in those areas; we must also search for new *L. major*-like strains, vectors, and hosts.
